# Physical Literacy and Resilience in Children and Youth

**DOI:** 10.3389/fpubh.2019.00346

**Published:** 2019-11-19

**Authors:** Philip Jefferies, Michael Ungar, Patrice Aubertin, Dean Kriellaars

**Affiliations:** ^1^Resilience Research Centre, Faculty of Health, Dalhousie University, Halifax, NS, Canada; ^2^Center for Research Innovation and Transfer in Circus Arts, National Circus School, Montreal, QC, Canada; ^3^Faculty of Health Sciences, University of Manitoba, Winnipeg, MB, Canada

**Keywords:** resilience, physical literacy, physical activity, physical education, children, youth, circus

## Abstract

**Background:** There is growing interest in the relationship between physical and psychosocial factors related to resilience to better understand the antecedents of health and successful adaptation to challenges in and out of school, and across the lifespan. To further this understanding, a trans-disciplinary approach was used to investigate the association between the multidimensional constructs of physical literacy and resilience in children at a key stage in their development.

**Methods:** Cross-sectional data were collected from 227 school children aged 9-12 years old from five schools in Winnipeg, Manitoba, Canada. Resilience was measured using the *Child and Youth Resilience Measure*, and physical literacy through the *Physical Literacy Assessment for Youth* tools. Data were provided by self-report, surrogate assessors of the child (physical education teachers and parents), and trained assessors for movement skills. These data were analyzed using correlation and logistic regression.

**Results:** Resilience was significantly correlated with numerous indicators of physical literacy, including movement capacity, confidence, and competence, environmental engagement, and overall perceptions of physical literacy. Regressions indicated that resilience could be predicted by movement confidence and competence, environmental engagement, and overall physical literacy.

**Conclusions:** The findings of this study, using a constellation of sources, provide foundational evidence for the link between resilience and physical literacy among children, encouraging the importance of physical literacy development in schools. Longitudinal studies are required to further examine this relationship and how these previously unrelated fields may work together for a richer understanding of the interplay between the physical and psychological determinants of well-being.

## Introduction

A multisystemic and social-ecological understanding of resilience asserts that young peoples' capacity to thrive despite exposure to adversity depends on the quality of their interactions with aspects of their environment, and the degree to which those environments provide the resources for the development or maintenance of optimal psychological, social, and physical well-being (1–3). This approach to resilience encourages the importance of the availability and accessibility of resources that can foster resilience, and that strengths in one domain may buffer against stressors related to those in another (2). For instance, studies have shown that school-based interventions designed to facilitate improvements in cognitive and interpersonal skills not only lead to better academic performance, but can also provide protective effects against social and health risks such as delinquency and psychosocial distress (4).

This has particular implications for approaches to create healthy schools, such as the Pan-Canadian Healthy School Planner (5) and the UK Resilience Programme (6), which adopts the Penn resilience programme used in the US (7). These approaches promote resilience-bolstering practices that focus on building *psychosocial* resources (e.g., self-awareness, self-management), but at present, neglect or demote the *physical* to physical education classes (or Daily Physical Activity, intramural physical activity programming, or recess), despite known links between physical and psychological domains [e.g., Deuster and Silverman (8)].

In a parallel field, physical literacy has been defined as the competence to perform movement skills and the knowledge, motivation, confidence, and understanding to value and take responsibility for engagement in physical activity across the lifespan (9, 10). It has also been described as the physical and psychological attributes that are foundational to participation in physical activity and therefore the capacity for an active lifestyle (11, 12). As the groundwork for physical activity, physical literacy is said to be the basis for sustaining the health of individuals (13, 14), and therefore maintaining a healthy workforce and reducing the burden on health systems (15, 16). This has led to suggestions that cultivating physical literacy is as essential as developing skills in literacy and numeracy (17, 18), and has prompted global interest, such as the UNESCO program focus (19) and part of the WHO's proposal to create “active societies” (20). In Canada and the United States, recognition of the importance of physical literacy and the limitations of traditional sports-based physical education have led to a shift to a holistic physical literacy enriched curricula, which nurtures skill, confidence, motivation, and participation (21–24).

**Figure 1:**
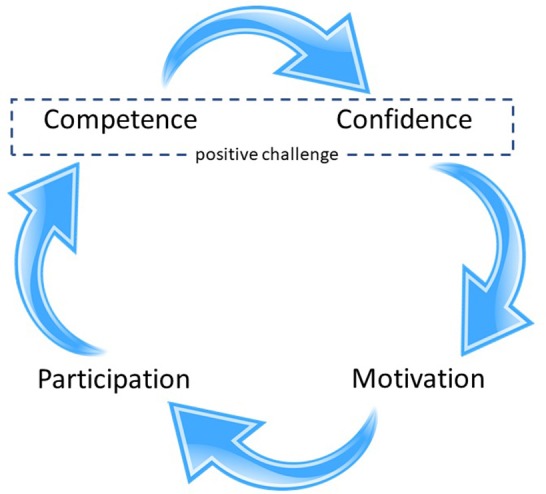
The cycle of physical literacy. Adapted from: Cairney et al. (14).

As physical literacy includes affective, social, and cognitive elements (9, 25), there are therefore commonalities with resilience. Furthermore, the core elements of both physical literacy and resilience develop when an environment is established which helps foster the ability to overcome challenges, obstacles, or adversity in physical and social settings (2, 3, 26). In the realm of resilience, this process of “steeling” suggests that limited exposure to adversity in appropriate environments can help an individual gain experience and coping strategies that can provide advantages in future encounters (27, 28), and has been linked to agency and persistence (29). Similarly, in physical literacy, the process of engaging in appropriately constructed challenges leads not only to improved basic movement competence, but concurrently the confidence and general competence to execute and assess movement in varied physical and social contexts, thus leading to the motivation for further physical activity engagement [see Figure 1, and for emerging evidence see Kriellaars et al. (30)]. The common process of engaging and overcoming challenges could be the basis for the association between the two constructs.

An investigation employing physical literacy could reveal whether the important foundational attributes for physical activity link to the established psychological and social factors related to resilience. Such a link would have implications for expanding school resilience programs to better support the well-being of students and trajectories for healthy development. It would also support the development of cross and cocurricular linkages and elevate the importance of physical education, as well as other movement-based school interventions. The objective of this study, then, was to investigate the association between physical literacy and resilience in children at a formative stage of their development. We hypothesized that measures of physical literacy would be significantly positively correlated with, and predictive of, resilience.

## Method

### Design

Baseline, cross-sectional data was extracted from a 3-year longitudinal project exploring the introduction of circus arts in the physical education (PE) curriculum in Canadian schools. This project was a partnership between the National Circus School, the Resilience Research Centre of Dalhousie University, Centre de liaison sur l'intervention et la prévention psychosociale (CLIPP), the Quebec federation of school boards (FCSQ), Concordia University, University of Manitoba, and the University of Alberta. Data were supplied by participants, as well as their PE teachers, their parents, and trained assessors, in order to gain insights from a range of informative and credible sources.

### Participants

Two hundred and twenty-seven elementary school children participated in the study (99 males, 122 females, 6 did not disclose) from five low-to-moderate socioeconomic status schools in Winnipeg, Manitoba. Students were in grades 4–6 with ages ranging from 9 to 12 years old (*M* = 10.04, *SD* = 0.47). Child assent and parental informed consent were obtained. Ethical approval for the study was granted by the University of Manitoba.

### Measures

Six separate assessments were included in the study. Five were from the Physical Literacy for Youth Assessment (PLAY) tools (31), designed to measure aspects of physical literacy from a variety of sources. Instructions for administration and further information can be found at physicalliteracy.ca/resources/.

The first assessment was the “PLAYfun,” which is completed by a trained assessor and measures an individual's competence of performing 18 curricular-linked movement skills (including running, locomotor movements, object control, balance, and stability), graded on a four-category 100 mm visual analog scale with 25 mm categories ranging from “initial,” “emerging,” “competent,” to “proficient” (α = 0.87) (32, 33). We used the average of the 18 skills as a measure of an individual's *movement competence*, and the sum as a measure of their *movement capacity*. The second tool was the “PLAYself” (7-day test-retest reliability = 0.83) (Jefferies et al., in preparation), which is completed by the child and includes a 12-item *physical literacy self-description*, rated on a four-point Likert scale (sample items include “I think I have enough skills to participate in all the sports and activities I want” and “I'm confident when performing activities”). It also includes a 6-item measure of *environmental engagement* (activity in and on water, on ice, on snow, and indoors and outdoors generally).

The third tool was the self-report “PLAYinventory” form (7-day test-retest reliability = 0.76), providing a count of the *number of activities* regularly participated in during the individual's leisure time over the last 12 months (out of over 80 possible activities). The fourth tool was the “PLAYparent” (7-day test-retest reliability = 0.73), completed by a parent to measure perceptions of their child's *movement competence* (6 items scored on a 3-point Likert scale) and their *overall physical literacy* (via a two-category 100 mm visual analog scale). The fifth tool was the “PLAYpe_teacher” (7-day test-retest reliability = 0.87), a modified version of “PLAYcoach,” providing measures of *movement competence* (6 items) and *confidence* (single item measure), both measured on a 5-point Likert scale ranging from “poor” to “excellent,” as well as the physical education teacher's rating of the student's *overall physical literacy* (via a two-category 100 mm visual analog scale). The tool also captures an individual's *overall fitness* and *activity level* (single item measures), measured on a 5-point Likert scale ranging from “poor” to “excellent,” in addition to their *body composition*—a “visual BMI”—involving a 10-picture body silhouette arranged in order of ascending BMI, which an individual is matched to (34, 35). These three fitness variables (measures associated with domain of physical activity) were included to investigate their possible relationship to resilience and to compare to the potential relationship between resilience and the physical literacy variables.

A novel sixth tool, also self-report, assessed resilience and peer relations, using the 12-item Child and Youth Resilience Measure (CYRM; α = 0.75) (36, 37) and the peer relations subscale of the Strengths and Difficulties Questionnaire (α = 0.60) (38). The CYRM is used to provide indications of an individual's social-ecological resilience, meaning that scores reflect the ability of individuals to engage with external resources in order to manage adversity. For ease of interpretation, the peer relations subscale was reverse-coded so that higher scores indicate better relations with peers.

### Data Collection

Data collection took place in October 2016 over a 2-week period, where the participants completed the PLAYself, PLAY Inventory, and the questionnaire containing the CYRM and peer relations scale. Eight trained assessors rated the participants on the PLAYfun using a similar approach to that of Kriellaars and colleagues (30), and PE teachers completed a PLAYpe_teacher assessment for each child based on their recall of an individual after 1 month of exposure in the school setting. Parents completed a PLAYparent assessment for their own children.

### Analyses

One-way ANOVAs were performed to explore differences between male and female participants, as these are often reported in the literature [e.g., O'Brien et al. (39)]. Correlational analyses were then performed to examine relationships between measures of physical literacy, resilience, and peer relations. Exploration of resilience scores suggested a threshold score of 29 was appropriate to dichotomise individuals into high (*n* = 139) and low (*n* = 53) categories, which corresponds with thresholds recommended for other versions of the measure (40). Given the interest in the identification and intervention of youth most in need of support, binary logistic regressions were employed to examine the capacity of physical literacy and fitness variables to predict whether individuals had high or low levels of resilience (linear regression using resilience as a continuous variable returned similar results but without classification information). All analyses were performed using SPSS v21 (41).

## Results

We discovered significant sex differences in trained assessor ratings of movement competence (*p* = 0.010) and capacity (*p* = 0.009), PE teacher ratings of movement confidence (*p* = 0.016), and overall activity level (*p* = 0.011). We also found significant differences between the sexes in parent perceptions of their child's movement competence (*p* = 0.022). In each instance, male participants scored significantly higher than their female counterparts (Table 1). No significant differences were observed between males and females among the remaining variables.

**Table 1:** Descriptive statistics and zero-order correlation coefficients for all variables against resilience scores.

**Source**	**Variable**	**Males (*n* = 99) Mean (*SD*)**	**Females (*n* = 122) Mean (*SD*)**	**Total sample Mean (*SD*)**	**Resilience r_**s**_**
Self-report	Resilience	30.38 (4.44)	30.21 (3.98)	30.30 (4.17)	–
Self-report	Peer relations	10.30 (1.95)	10.32 (1.92)	10.32 (1.93)	0.31^**^
Self-report	No. of activities	26.72 (13.15)	25.83 (11.09)	26.24 (11.99)	0.03
Self-report	PL: Environmental engagement	24.39 (3.40)	23.90 (3.30)	24.13 (3.33)	0.22^**^
Self-report	PL: PL self-description	33.94 (4.96)	33.44 (5.59)	33.66 (5.28)	0.30^**^
Trained assessor	PL: Movement competence	33.44 (7.17)	30.76 (7.39)	31.99 (7.39)	0.16^*^
Trained assessor	PL: Movement capacity	601.26 (129.34)	551.85 (133.86)	574.47 (133.79)	0.15^*^
Parent	PL: Overall rating	23.29 (4.03)	22.79 (3.06)	23.07 (3.56)	0.14
Parent	PL: Movement competence	16.37 (2.85)	15.31 (2.10)	15.85 (2.54)	0.08
PE teacher	PL: Movement confidence	18.52 (4.46)	16.96 (4.72)	4.19 (1.73)	0.25^**^
PE teacher	PL: Movement competence	24.74 (6.72)	23.77 (5.98)	24.25 (6.34)	0.17^**^
PE teacher	PL: Overall rating	4.27 (1.77)	4.10 (1.70)	17.69 (4.68)	0.21^*^
PE teacher	Fitness	3.47 (1.00)	3.20 (1.13)	3.33 (1.08)	0.26^**^
PE teacher	Activity level	3.60 (0.92)	3.22 (1.13)	3.40 (1.06)	0.23^**^
PE teacher	Body composition	2.83 (1.73)	2.95 (2.27)	2.90 (2.03)	−0.06

* *p ≤ 0.05*,

** *p ≤ 0.001. PL, physical literacy; PE, physical education*.

As hypothesized, resilience scores were positively correlated with trained assessor ratings of movement competence (*r*_*s*_ = 0.16, *p* = 0.031) and capacity (*r*_*s*_ = 0.15, *p* = 0.042), as well as PE teacher ratings of competence (*r*_*s*_ = 0.17, *p* = 0.016), confidence (*r*_*s*_ = 0.25, *p* = 0.001), overall physical literacy (*r*_*s*_ = 0.21, *p* = 0.004), fitness (*r*_*s*_ = 0.26, *p* < 0.001), and activity levels (*r*_*s*_ = 0.23, *p* = 0.001). Resilience was also positively correlated with self-reported physical literacy (*r*_*s*_ = 0.30, *p* < 0.001) and environmental engagement (*r*_*s*_ = 0.22, *p* = 0.001). Parental ratings of movement competence and overall physical literacy, body composition, and number of self-reported activities were not found to be related to resilience.

Resilience scores also positively correlated with peer relations (*r*_*s*_ = 0.31, *p* < 0.001). Additionally, peer scores correlated with PE teacher assessments of confidence (*r*_*s*_ = 0.15, *p* = 0.036), movement competence (*r*_*s*_ = 0.14, *p* = 0.050), overall physical literacy (*r*_*s*_ = 0.16, *p* = 0.033), fitness (*r*_*s*_ = 0.27, *p* < 0.001) and activity levels (*r*_*s*_ = 0.18, *p* = 0.015), but unlike resilience scores, peer scores negatively correlated with bodily composition (*r*_*s*_ = −0.18, *p* = 0.015), indicating that higher BMIs were associated with lower peer scores. Additionally, unlike resilience, peer scores were not found to correlate with assessor ratings of movement competence or capacity, nor self-reported physical literacy or environmental engagement.

We then performed a binary logistic regression, and a backwards stepwise approach involving all physical literacy variables revealed a significant and parsimonious solution at the sixth step ($\chi_{5}^{2}$ = 23.731, *p* < 0.001). The model, accounting for 33% of the variance in resilience scores (Nagelkerke R^2^), correctly predicted 79% of cases (1.2% more than steps 5 and 7) and involved PE teacher perceptions of movement confidence and competence, parent perceptions of movement competence, and self-reported environmental engagement and perceptions of physical literacy. Numerous models using physical literacy variables were achievable. However, when adding fitness, activity level, and body composition in a second block of predictors, the model was also significant ($\chi_{6}^{2}$ = 24.101, *p* = 0.001), but these variables did not add predictive value (79% of cases correctly predicted, 33% of variance accounted for). Finally, we performed the binary logistic regression using the fitness, activity, and body composition variables alone, and a significant model was not produced.

## Discussion

Both the correlative and predictive results of the study demonstrate a clear association between resilience and physical literacy in children, arising from a constellation of sources, involving self-perception, perception of a teacher, or assessment by trained observers. Specifically, we found that an individual's resilience is associated with their competence and confidence to move, which are the key components of physical literacy (9). Only parent perceptions did not correlate with self-reported levels of resilience, which may be explained by the tendency for parents to overestimate the abilities of their children (42, 43). Additionally, no sex differences were detected in self-reports of resilience, but were found in physical literacy variables such as movement confidence, which were significantly higher in male participants compared to females, fitting with the literature (39, 44, 45).

To our knowledge, this is the first study to demonstrate the connection between resilience and physical literacy. Although the mechanism which links resilience and physical literacy remains unclear, it presents the beginning of an important transdisciplinary approach (46, 47). For instance, if the affective domains of confidence and motivation developed in physical literacy (25) go beyond just motor action, then they may also provide or help young people acquire the skills and abilities to better negotiate for, and navigate to, resources that sustain their well-being in different contexts. This is fundamental to resilience (48). Furthermore, the contexts in which movement competence develops are both physical (land, air, ice, snow, water) and sociocultural, and require that these competencies are developed with exposure to problem-solving settings in both (26). This exposure to and mastery of ‘positive challenge’ may position physical literacy as an antecedent of resilience. Our study is cross-sectional and therefore prohibits establishing causal mechanisms; however, either direction of action is beneficial, as improvements in resilience may lead to greater confidence and motivation in physical activity, and therefore to greater participation, health, and well-being (8). A longitudinal study is required to examine the causal couplings between the constructs.

These findings respond to the emerging interest in how physical literacy relates to health (20) and the link to mental, physical and social well-being. Our study supports the notion that encouraging physical literacy is associated with fostering resilience, and so creating the underlying conditions for individuals to thrive and participate actively in society. This has important implications for curricula development in schools. For instance, by including physical literacy development, and so acknowledging the importance of physical as well as psychosocial factors associated with resilience, such a holistic reframing broadens access to the resources for young people to thrive. Furthermore, although there have been resilience-related investigations into the protective factors and stressors involved in sport (49, 50), the study of physical literacy is broader and likely more relevant to vulnerable persons that may not be engaged in traditional sports activities; physical literacy includes movement in vocation, recreation, performance arts, and school, as well as sport (51). Therefore, the inclusion of robust physical literacy curricula in schools would likely lead to a greater participatory culture and arguably to greater human and social equity [see the “power pillar” of physical literacy (26)]. Indeed, some health and physical education curricula have already adopted a physical literacy approach (22, 24), and initial implementations have been highly successful (30).

Other findings in this study point to interesting areas of further research. For example, while peer relations were not associated with most sources of physical literacy, we found that they were associated with PE teacher ratings of elevated BMI and other physical literacy-related variables. This may indicate a separation of the factors implicated in peer relationships and resilience. In a social-ecological model of resilience which emphasizes the combined impact of psychological, social, cultural, and physical resources that sustain well-being (2, 48), peer relationships may be only one dimension of resilience as it links to physical literacy. However, as both peer relationships and body composition are known to impact resilience (48, 52), it is also possible that some resilience and physical literacy variables may begin to interact at a later age. This is encouraged by our findings reflecting the literature that fitness, activity level, and body composition were associated with each other (53) and with physical activity (54), but that they did not add to the predictive capacity of our model. Perhaps at 9–12 years old (the age of our participants) these factors may not yet have an impact on resilience, but as children move into older adolescence, levels of fitness, activity, body composition, and peer relationships become more important. Process models of physical literacy (14, 55) propose a positive feedback cycle of motivation and confidence which leads to competence (and then participation); a process which may begin pre-adolescence and become more strongly associated with resilience in adolescence [see the “journey pillar” of physical literacy (26)]. As this was an exploratory cross-sectional study, we cannot confirm these relationships, however, the study stimulates thinking about the conceptualization of physical literacy and resilience as processes that are likely interactive.

As this study is part of a longitudinal project, we will be able to track variations in resilience and physical literacy to shed light on how changes in each interact from pre-adolescence into adolescence. Such a study would be particularly valuable given the precipitous drop in physical activity that occurs around age 12 (56), and potentially inform whether the causal factors are pre-pubescent or related to the pubescent transition itself. This line of further investigation may also explain why no sex difference was found for self-reported resilience in the current study, but female participants scored more poorly than males on a number of physical literacy indicators. As physical activity levels decrease more steadily for females than for males into adolescence, investigating corresponding changes in resilience could also help to address the challenge related to sex-related differences in resilience that have been identified at this age (57–59). This may then point to critical ages for schools to invest in physical literacy development to reduce the gap in movement competence and confidence (30).

## Conclusion

This study directly links resilience and physical literacy, bringing together two previously distinct approaches to the well-being of young people and confirming their relationship. Our findings support the value of physical literacy development in schools as part of a holistic approach toward supporting the well-being of young people and their future health. Further longitudinal research into the interaction of resilience and physical literacy processes could provide insight into creating optimal physical-psycho-social environments in schools that foster the development of both constructs, and the possible interlinking to other key processes, such as creativity.

## Data Availability Statement

The raw data supporting the conclusions of this manuscript will be made available by the authors, without undue reservation, to any qualified researcher.

## Ethics Statement

The studies involving human participants were reviewed and approved by University of Manitoba Research Ethics Board. Written informed consent to participate in this study was provided by the participants' legal guardian/next of kin.

## Author Contributions

MU, PA, and DK conceived of the project and contributed to the revising and editing of the manuscript. DK and PJ aided in collecting and analyzing the data, and to the writing and revising of the manuscript. All authors reviewed and approved the manuscript.

### Conflict of Interest

The authors declare that the research was conducted in the absence of any commercial or financial relationships that could be construed as a potential conflict of interest.
